# CNS Pericytes Modulate Local T Cell Infiltration in EAE

**DOI:** 10.3390/ijms232113081

**Published:** 2022-10-28

**Authors:** Kathrin Koch, Maren Lindner, Ann-Katrin Fleck, Marie Liebmann, Melanie Eschborn, Lisa Zondler, Rodrigo Diéguez-Hurtado, Ralf H. Adams, Gerd Meyer zu Hörste, Alexander Zarbock, Tanja Kuhlmann, Heinz Wiendl, Luisa Klotz

**Affiliations:** 1 Department of Neurology with Institute of Translational Neurology, University Hospital Muenster, 48149 Muenster, Germany; 2 Department of Anaesthesiology, Intensive Care and Pain Medicine, University Hospital Muenster, 48149 Muenster, Germany; 3 Department of Tissue Morphogenesis, Faculty of Medicine, Max Planck Institute for Molecular Biomedicine, University of Muenster, 48149 Muenster, Germany; 4 Institute of Neuropathology, University Hospital Muenster, 48149 Muenster, Germany

**Keywords:** pericytes, blood–brain barrier, experimental autoimmune encephalomyelitis, multiple sclerosis, antigen presentation, central nervous system

## Abstract

Pericytes at the blood–brain barrier (BBB) are located between the tight endothelial cell layer of the blood vessels and astrocytic endfeet. They contribute to central nervous system (CNS) homeostasis by regulating BBB development and maintenance. Loss of pericytes results in increased numbers of infiltrating immune cells in the CNS in experimental autoimmune encephalomyelitis (EAE), the mouse model for multiple sclerosis (MS). However, little is known about their competence to modulate immune cell activation or function in CNS autoimmunity. To evaluate the capacity of pericytes to directly interact with T cells in an antigen-specific fashion and potentially (re)shape their function, we depleted major histocompatibility complex (MHC) class II from pericytes in a cell type-specific fashion and performed T cell-pericyte cocultures and EAE experiments. We found that pericytes present antigen in vitro to induce T cell activation and proliferation. In an adoptive transfer EAE experiment, pericyte-specific MHC II KO resulted in locally enhanced T cell infiltration in the CNS; even though, overall disease course of mice was not affected. Thus, pericytes may serve as non-professional antigen-presenting cells affecting states of T cell activation, thereby locally shaping lesion formation in CNS inflammation but without modulating disease severity.

## 1. Introduction

Pericytes are capillary-associated mural cells, which control microvascular functions, such as limiting vascular permeability, and are essential for maintaining CNS homeostasis by preserving BBB integrity and cerebral blood flow [1,2,3,4,5]. Recent publications demonstrated that pericyte loss is linked to many neurodegenerative diseases, including Alzheimer’s disease (AD) [6,7], mild dementia [8], stroke [9,10], and cerebral autosomal dominant arteriopathy with subcortical infarcts (CADASIL) [11]. Additionally, pericytes participate in the regulation of leukocyte migration across barriers in different organs [12,13,14,15]. For instance, pericytes of the CNS control leukocyte infiltration during homeostasis and autoimmune neuroinflammation [16], in particular in multiple sclerosis (MS). The pathogenesis of MS is characterized by autoreactive immune cells targeting structures of the myelin sheet in the CNS and subsequently leading to inflammatory lesion formation, temporal break-down of the BBB integrity, demyelination, and axonal damage [17]. Antigen-presenting cells (APC) play a critical role in the peripheral priming of myelin-specific T cells as well as in their reactivation while or after entering the CNS [18,19,20,21]. Since pericytes are presumed to contribute to the regulation of BBB permeability in MS [16,22] as well as in remyelination of MS lesions [23], we investigated whether brain pericytes are involved in the antigen presentation process during experimental autoimmune encephalomyelitis (EAE). To address this, we made use of a pericyte-specific Cre-inducible mouse model to ablate MHC II on the pericyte cell surface. Here, we showed that pericytes are capable of presenting antigens in vitro to modulate T cell proliferation and activation. MHC II depletion on pericytes in vivo did not affect the overall disease course but resulted in reduced infiltration of pro-inflammatory CD4^+^ T cells in the CNS when compared to MHC II wild-type animals during EAE.

## 2. Results

**Figure 1:**
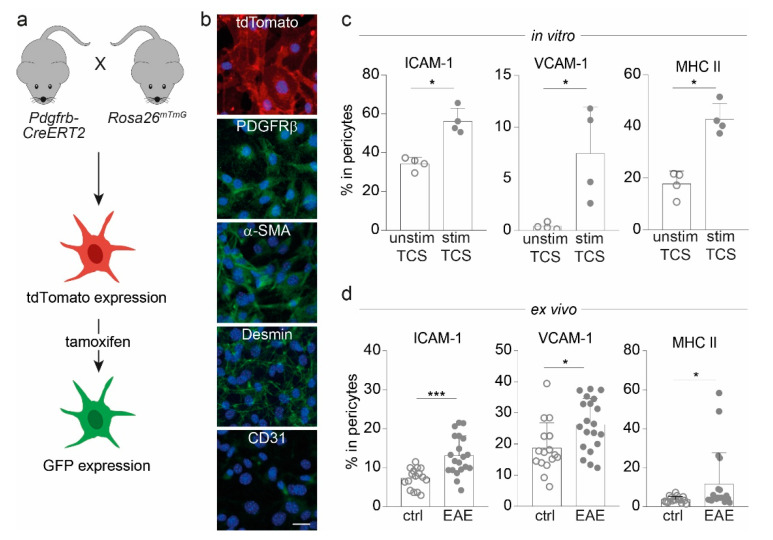
Pericytes from *Pdgfrb-CreERT2-GFP* mice express molecules important for cell adhesion and antigen presentation. (**a**) Overview of *Pdgfrb-CreERT2-GFP* mice with tdTomato-labeled pericytes, which express GFP upon tamoxifen administration. (**b**) Images of cortical pericytes stained with immunofluorescent antibodies depict the expression of tdTomato, typical mural cell markers (PDGFRβ, α-SMA and desmin), and CD31 as endothelial cell marker (scale bars = 50 µm). (**c**) Bar graphs show the frequencies of ICAM-1, VCAM-1 and MHC II positive cells in pericytes treated with T cell supernatant (TCS) either from 72 h-stimulated or unstimulated T cells in vitro after 72 h assessed via flow cytometry. (**d**) Frequencies of ICAM-1, VCAM-1, and MHC II-expressing pericytes isolated from the CNS of *Pdgfrb-CreERT2-GFP* animals immunized with MOG_35–55_ at disease maximum (EAE; *n* = 20) in comparison to non-immunized mice (ctrl; *n* = 16) are illustrated. All data depict mean ±SD. Whereas one dot represents for (**c**) one individual experiment and for (**d**) one dot represents one individual mouse. Data shown are from at least three independent experiments. Statistical analysis was performed using Mann–Whitney U-test. *, *p* ≤ 0.05; ***, *p* ≤ 0.001.

For investigating pericytes in the context of antigen-specific interaction with CD4^+^ T cells and their subsequent effects in the context of CNS autoimmunity, *Pdgfrb-CreERT2* mice were crossbred with *Rosa26mTmG* reporter animals (hereafter named *Pdgfrb-CreERT2-GFP*). This approach has proven valuable for targeting and detection of pericytes [24] based on their expression of platelet-derived growth factor receptor beta (PDGFRβ) (Figure 1a). First, we isolated cortical pericytes from these mice without previous tamoxifen administration and verified the expression of the tdTomato signal and classical mural cell markers, e.g., PDGFRβ, alpha-smooth muscle actin (α-SMA), and desmin, while the cells were negative for the endothelial cell marker CD31 (Figure 1b). Next, we examined the expression of immunologically relevant molecules via flow cytometric analysis of these cells upon in vitro stimulation. Pericytes treated with T cell supernatant (TCS) derived from stimulated CD4^+^ T cells showed a significant increase in the expression of adhesion molecules, such as intracellular adhesion molecule (ICAM-1) and vascular cell adhesion molecule (VCAM-1) as well as MHC II compared to pericytes treated with TCS from unstimulated T cells (Figure 1c). This increase of adhesion molecules and MHC II was also observed ex vivo in pericytes isolated from the cortices of EAE mice compared to non-immunized controls (Figure 1d).

**Figure 2:**
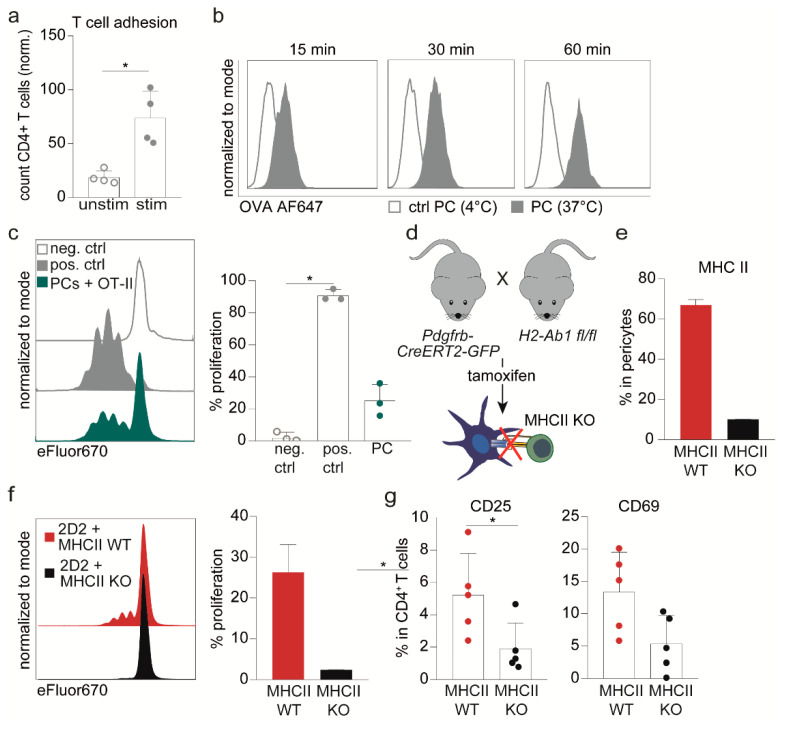
Pericytes activate T cells in an antigen-specific manner. (**a**) Bar graph depicts the quantification of the adhesion of stimulated or unstimulated CD4^+^ T cells to pericytes evaluated via immunofluorescence. (**b**) Histograms show the uptake of Alexa Fluor 647-labeled OVA protein at 37 °C and at 4 °C (as control) by pericytes at indicated time points. (**c**) Proliferation assessed by flow cytometric analysis of antigen-specific activated OT-II CD4^+^ T cells after 72 h co-cultivation with pericytes (PCs) loaded with specific OVA peptide (OVA_323–339_). As positive control (pos. ctrl) DC loaded with specific OVA peptide were used while unstimulated cells were used as negative control (neg. ctrl.). Depicted are representative proliferation profiles (left) and percentages of proliferated T cells (right, *n* = 3). (**d**) Scheme of the *Pdgfrb-CreERT2-MHCII* mouse model. Tamoxifen administration leads to depletion of MHC II on pericytes in Cre^+^ littermates, whereas pericytes in Cre^−^ littermates remain unaffected. (**e**) Bar graph depicts representative frequencies of MHC II-expressing pericytes after 72 h TCS stimulation of in vitro Cre-recombined (MHC II KO) or untreated (MHC II WT) cortical pericytes of *Pdgfrb-CreERT2-MHCII* mice (*n* = 2). (**f**) Proliferation of 2D2 CD4^+^ T cells is shown after co-cultivated for 72 h with either MHC II WT or MHC II KO pericytes loaded a particular neurofilament (NFM_15–35_) peptide. Depicted are representative proliferation profiles (left) and percentages of proliferated T cells (right, *n* = 2). (**g**) Dot plots depict the frequencies of 2D2 CD4^+^ T cells expressing early activation marker CD25 and CD69 when co-cultivated with NFM_15–35_-loaded MHC II KO pericytes compared to WT pericytes after 48 h. Data from (**a**,**c**,**g**) depict mean ± SD of at least three independent experiments. Single dots represent values of one individual experiment. (**b**,**e**,**f**) represent on exemplary experiment out of four different experiments. Statistical analysis was performed using Kruskal–Wallis Test for three groups (c; one-way ANOVA) and Mann–Whitney U-test for two groups (**g**). * *p* ≤ 0.05.

As pericytes can interact with peripheral immune cells in vivo, e.g., at the cremaster muscle [13], we were interested in whether such an interaction may also occur between CD4^+^ T cells and brain pericytes. As a first step, we performed a static adhesion experiment in vitro and detected a significant increase in the adherence of stimulated CD4^+^ T cells to pericytes in comparison to unstimulated CD4^+^ T cells (Figure 2a). Since pericytes upregulated MHC II upon stimulation, we next investigated whether they presented antigens to adherent T cells. Antigen uptake experiments revealed that pericytes take up the model antigen ovalbumin (OVA) in a time-dependent fashion (Figure 2b). Notably, OVA-peptide-loaded pericytes were presented antigen towards CD4^+^ T cells and elicited T cell proliferation upon interaction, although professional APC, e.g., dendritic cells (DC), were more efficient (Figure 2c). In this context, also low expression of costimulatory molecules, e.g., CD40, CD80, and CD86 were noticed on pericytes (Supplementary Figure S1a).

To validate the relevance of MHC II for T cell activation, we developed a conditional knock-out mouse model in which MHC II can be depleted in *Pdgfrb*^+^ cells upon tamoxifen administration (Figure 2d). In isolated brain pericytes of these *Pdgfrb-CreERT2-MHCII* floxed mice (henceforth termed MHC II KO), KO cells reach only 20% of MHC II expression compared to WT pericytes (Figure 2e). Indeed, this targeted ablation of MHC II was accompanied by a complete loss of the ability of pericytes to prime naive CD4^+^ T cells in vitro, as illustrated by the absence of T cell proliferation (Figure 2f) and the reduced expression of activation markers such as CD25 and to a lesser extent CD69 (Figure 2g). 

**Figure 3:**
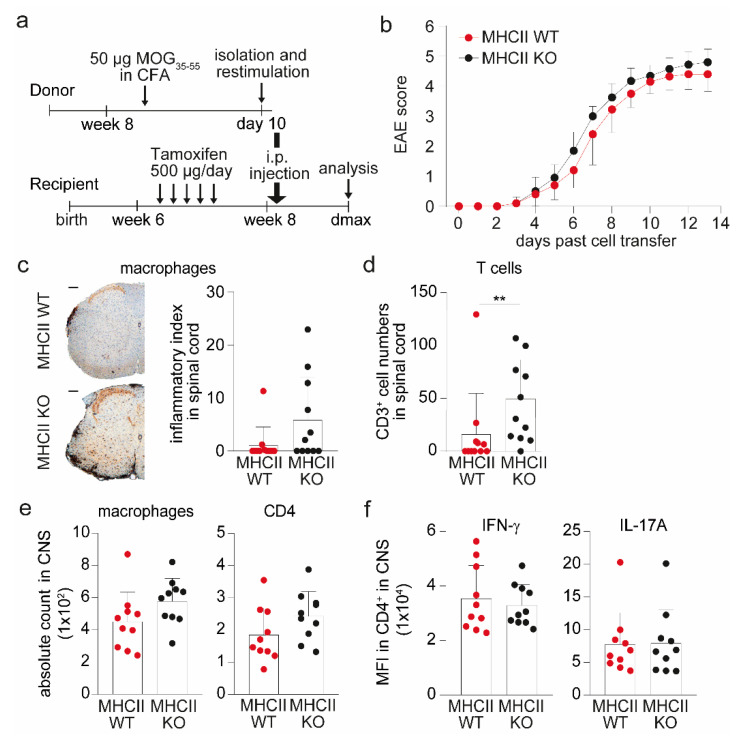
Pericytes control local T cell infiltration in an MHC II-dependent manner during AT-EAE. (**a**) Schematic overview of AT-EAE experiments in *Pdgfrb-CreERT2-MHCII* animals is shown. (**b**) Disease course of one AT-EAE experiment is illustrated as a clinical score of MHC II WT (*n* = 10) compared to MHC II KO mice (*n* = 10). (**c**) Histological analysis was performed with regard to cell infiltration into the spinal cords. Depicted are representative images of histological sections stained with MAC3^+^ to evaluate macrophage infiltration into the spinal cord of MHC II WT mice and MHC II KO animals (left, scale bar = 200 µm). Quantification of the staining is illustrated as inflammatory index, which is calculated as the ratio between the area of infiltrated macrophages and the area of white matter (right, *n* = 11 mice/group). (**d**) Bar graph depicts the mean count of CD3^+^ T cells in the spinal cords of MHC II KO (*n* = 11) mice compared to MHC II WT (*n* = 11) mice by histological analysis. (**e**) Absolute cell counts of infiltrated macrophages and CD4^+^ T cells in the CNS of *Pdgfrb-CreERT2-MHCII* animals evaluated via flow cytometry are shown. (**f**) Bar graphs depict the mean fluorescence intensities (MFI) of pro-inflammatory cytokines IFN-γ and IL-17A in CD4^+^ T cells isolated from the CNS of MHC II KO or MHC II WT animals. All data are shown as mean ± SD. Single dots represents individual values of single mice. Statistical analysis was performed using Mann–Whitney U-test. ** *p* ≤ 0.01.

Finally, we wondered whether targeting antigen-specific interactions between pericytes and CD4^+^ T cell via MHC II ablation in pericytes might have an impact on the course of CNS autoimmunity in vivo. To focus on potential pericyte—CD4^+^ T cell interactions during T cell entry into the CNS and exclude peripheral T cell priming effects, we made use of the adoptive transfer model of experimental autoimmune encephalomyelitis (AT-EAE). Transfer of in vitro restimulated wild-type T cells from MOG-immunized MHC II WT animals either into MHC II KO or MHC II WT animals after tamoxifen application (Figure 3a) revealed a similar disease course in both groups (Figure 3b). In semi quantitative immunohistochemistry of spinal cord sections, a tendency towards a larger area of infiltrated macrophages was observed in MHC II KO animals (Figure 3c). In this line, MHC II KO mice displayed significantly increased CD3^+^ T cells numbers compared to WT animals (Figure 3d). Using Evans Blue injection, concomitant effects on BBB integrity mediated by the knock-out of MHC II were excluded (Supplementary Figure S1b).

In contrast to the histological assessment, flow cytometric analysis of whole CNS lysates, combining spinal cords and brain, did not reveal significantly altered numbers of macrophages and CD4^+^ T cells (Figure 3e), thus pointing towards local differences within the lesions as opposed to general differences between both groups with regard to immune cell infiltration into the CNS of MHC II KO versus WT mice. Furthermore, no difference in the activation status of CNS-isolated CD4^+^ T cells was detected, as measured by the pro-inflammatory cytokine production of IFN-γ and IL-17A (Figure 3f). Similar to the AT-EAE model, no differences in disease severity were observed in an active EAE experiment when comparing MHC KO versus WT mice (Supplementary Figure S1c).

Taken together, our data suggest that pericytes are indeed capable of specifically interacting with CD4^+^ T cells and activate them in an antigen-specific fashion, in particular under inflammatory conditions resulting in upregulation of key molecules involved in immune cell adhesion and antigen-presentation. In the context of CNS autoimmunity, this might locally modulate T cell infiltration into the CNS but is dispensable for disease outcome. 

## 3. Discussion

In recent years, extensive research on the complex functions of the BBB revealed that the migration of circulating immune cells is required for the maintenance of CNS immune surveillance even under homeostatic conditions [25]. In addition, the regulatory functions of other BBB components have also become a focus of research, including the role of basement membrane components such as laminins and the role of pericytes in immune cell transmigration across the BBB [16,26,27]. In the context of autoimmunity, the potential role of pericytes remains to be fully elucidated. In a recent study, pericyte-deficient mice exhibit an aggravated and atypical course of experimental autoimmune encephalitis, suggesting that pericytes may indeed modulate immune cell entry into the CNS. However, the underlying molecular mechanisms shaping such an interaction have not been addressed in this context [16]. Another publication described an increase in pericyte vessel coverage in the context of CNS inflammation and suggested that pericytes might be involved in macrophage recruitment into the CNS during EAE [27]. In human MS lesions, it has been demonstrated that mesenchymal perivascular cells, which share common markers with pericytes, express HLA-DR in inflammatory active lesions, at least suggesting that pericytes might be involved in the presentation of antigens towards CD4^+^ T cells [22]. 

Here, we show that CNS pericytes express several immune-regulatory molecules relevant for shaping T cell functions and interactions in the context of CNS autoimmunity, e.g., by priming naïve T cells in an antigen-specific fashion. Although the extent of T cell activation elicited by pericytes was not as strong as the one by professional APCs, such as DCs. Similar results were demonstrated for other so-called non-professional APCs, such as hepatic stellate cells, liver sinusoidal endothelial cells as well as lung fibroblasts [28,29,30].

However, in contrast to in vitro priming experiments in the context of CNS autoimmunity, cellular components of the BBB may be preferentially involved in attracting and reactivating autoreactive T cells that have already been primed in the periphery. Therefore, we investigated the functional relevance of the antigen presentation of brain pericytes towards antigen-experienced CD4^+^ T cells employing pericyte-specific MHC II KO animals in the AT-EAE model upon transfer of already primed CD4^+^ T cells [31]. Indeed, histological analysis revealed increased CD4^+^ T cell numbers in the spinal cord lesions of MHC II KO animals, indicating that antigen-specific interactions of pericytes with CD4^+^ T cells might influence lesion formation in the CNS. Comparing these results of inflammatory lesion composition with our flow cytometry data of whole CNS tissue, it might be surprising that the latter approach revealed no differences in the frequency of activated CD4^+^ T cells in the CNS; however, it has to be taken into account that our flow cytometric approach was performed in pooled spinal cord and brain homogenates, therefore potentially masking more subtle local differences in lesion composition by this approach. In our view, this lesion-specific difference in T cell infiltration in MHC II KO brains points towards a targeted antigen-specific influence of pericytes on T cell interaction and lesion development, whereas the extent of overall inflammation in this particular EAE model is not influenced by pericyte specific ablation of MHC class II expression. 

Surprisingly, we observed increased T cell numbers in inflammatory lesions of MHC II KO mice compared with MHC II WT animals, indicating that pericyte-specific presentation of (auto-)antigens towards T cells tends to control subsequent T cell invasion into the surrounding tissue, thereby potentially attenuating T cell responses within the CNS. Similarly, suppressive functions of pericytes in the vicinity of tumor tissue were described to control CD4^+^ T cell activation and elicit antigen-specific T cell anergy [32]. Our observation is in line with the recent observation that absence of pericytes at the BBB results in increased immune cell infiltration into the CNS during EAE, again pointing to a gatekeeping function of pericytes controlling the extent of immune cell infiltration in the context of CNS inflammation [16]. Our study at least suggests that antigen-specific interaction with encephalitogenic T cells via MHC class II might be involved in this process.

As shown for other non-professional APCs, control of T cells might be associated with lower expression levels of costimulatory molecules [29]. Indeed, analysis of costimulatory molecules on pericytes in EAE revealed low expression levels of CD40, CD80 and CD86, supporting the concept that antigen-specific interaction of pericytes and CD4^+^ T cells together with weak costimulatory signals limits CD4^+^ T cell activity. Notably, to rule out any impairment of BBB integrity by MHC II KO, we injected Evans Blue and did not observe an altered barrier integrity in MHC II KO mice.

EAE models, especially AT-EAE models, are far from representing physiological conditions because T cell activation is highly artificial, and the stimulation approaches used are very strong [33]. Furthermore, the tamoxifen administration in the recipient mice might also impact the immune response and therefore the EAE disease course in this model system. The fact that alteration of the antigen presentation capacity via MHC II by pericytes had no effect on overall disease severity in EAE does not rule out any physiological role of antigen-presenting pericytes in other conditions, such as acute or chronic viral infections, mechanisms involved in CNS immunosurveillance or more subtle and chronic autoimmune reactions, such as in human MS. Indeed, our results together with other studies [16,27] illustrate that pericytes have the capacity to directly influence immunological processes in the CNS, both by modulating brain barrier integrity and by directly interacting with autoreactive T cells. Therefore, it is reasonable to assume that pericytes also guide immune cell entry into the CNS tissue and shape T cell functions during this process. 

Finally, it cannot be excluded that recombination efficiencies may vary in the tamoxifen-inducible system that was used, even if in our *Pdgfrb*-reporter mice recombination appears convincing (Supplementary Figure S2a) and we applied tamoxifen to both groups to overcome potential influences on the peripheral immune response. However, other studies showed recombination efficiencies of about 80% in the brains of adult *Pdgfrb*-reporter mice [24]. Furthermore, other *Pdgfrb*-expressing cells, such as fibroblasts and vascular smooth muscle cells, may also be targeted by *Pdgfrb* as a promoter for the mouse model, and this may in principle also potentially affect interactions with T cells [34,35,36]. Even though, by using the AT-EAE model, where already primed T cells are transferred into MHC II KO mice, at least a potential effect on peripheral T cell priming, as opposed to active EAE, can be largely excluded.

Taken together, our data point towards a role of CNS pericytes in shaping CD4^+^ T cell functions during their transmigratory process at the BBB upon antigen-specific interaction. Further studies are needed to decipher the role of pericytes in the context of chronic inflammatory diseases such as human MS.

## 4. Materials and Methods

### 4.1. Mice and Inducible Genetic Experiments

All strains were bred on a C57BL/6 background. *Pdgfrb(BAC)-CreERT2* [36] mice cross bred with *Rosa26^mTmG^* [37] were a kind gift of Prof. Ralf. H. Adams and are further referred to as *Pdgfrb-CreERT2-GFP* animals. The pericyte-specific MHC II KO animals were generated by crossbreeding *Pdgfrb-CreERT2-GFP* mice with *H2-Ab1* floxed mice from the Jackson Laboratory (Stock No: 013181) and are further referred to as *Pdgfrb-CreERT2-MHCII* mice. All mice were maintained under conditions of individually ventilated cages at the local animal facility of the University of Muenster. All animal studies were performed according to the guidelines of the animal ethics committee and were approved by the governmental authorities (84-02.04.2013.A017 and 81-02.04.2019.A488). Cre-mediated recombination was induced in 6–8-week-old mice by intraperitoneal (i.p.) injection of 500 μg of tamoxifen (Sigma-Aldrich, St. Louis, MO, USA; T5648; dissolved in ethanol-peanut oil (Sigma-Aldrich, St. Louis, MO, USA; P2144) 1/32 at 5 mg/mL) on 5 consecutive days.

### 4.2. Immunization of Mice for EAE

*Pdgfrb-CreERT2-GFP* animals as well as Cre^−^ and Cre^+^ littermates of *Pdgfrb-CreERT2-MHCII* mice were immunized as described previously [31]. Briefly, mice were immunized with 50 µg myelin oligodendrocyte glycoprotein (MOG_35–55_) peptide (generated by Rudolf Volkmer, Charite Berlin, Germany) emulsified in complete Freund’s adjuvant (Difco by Becton, Dickinson and Company (BD), Sparks, MD, USA; 263810) including *Mycobacterium tuberculosis* (Difco by BD, Sparks, MD, USA; 231141) by subcutaneous (s.c.) injection. On days 0 and 2 mice also obtained 300 ng of *Bordetella pertussis* toxin (PTx) (Sigma-Aldrich, St. Louis, MO, USA; P2980) i.p.

### 4.3. Adoptive Transfer Experimental Autoimmune Encephalomyelitis (AT-EAE)

Donor mice (Cre^−^ littermates of *Pdgfrb-CreERT2-MHCII* mice) were immunized with 50 µg MOG_35–55_ peptide (generated by Rudolf Volkmer, Charite Berlin, Germany) emulsified in complete Freund’s adjuvant (Difco by BD, Sparks, MD, USA; 263810) including *Mycobacterium tuberculosis* (Difco by BD, Sparks, MD, USA; 231141) by s.c. injection. After 10 days immune cells from lymph nodes and spleen were isolated and in vitro re-stimulated for 72 h in the presence of 20 mg/mL MOG_35–55_ and 20 ng/mL IL-12 (Peprotech, Hamburg, Germany; 210-12). Cells (5 × 10^6^) were injected i.p. into tamoxifen-treated recipient mice (Cre^−^ mice = MHC II WT, Cre^+^ mice = MHC II KO).

### 4.4. Disease Scoring

Starting at the day of the cell transfer or immunization, mice were clinically scored daily. Disease severity was determined using a scale from 0 to 8 as described previously [31]: 0, healthy; 1, limp tail; 2, weak unilateral paresis of hindquarters; 3, severe unilateral or weak bilateral paresis of hindquarters; 4, moderate bilateral paresis of hindquarters; 5, complete paralysis of hindquarters; 6, plegia of hindquarters; 7, quadriplegia; and 8, death. According to animal welfare, mice were sacrificed when scored 6 to minimize the extent of burden.

### 4.5. Evans Blue Injection

The integrity of the BBB of *Pdgfrb-CreERT2-MHCII* mice was verified by injection of Evans blue. The dye was dissolved in PBS at a concentration of 2% *w*/*v* and the solution was injected intravenously (i.v.) into the tail vein of each mouse and was allowed to circulate for 60 min. Afterwards, the mice were perfused and spinal cords as well as brains were prepared for photographing, using an acrylic brain matrix for 2-mm-thick sections. The CNS sections were photographed to detect and record the leakage of the dye into the CNS parenchyma.

### 4.6. Isolation and Culture of Cortical Brain Pericytes

The isolation of cortical pericytes was performed similarly as described previously Tigges et al., 2012 [38]. In detail, cortices from 6–12-week-old mice of *Pdgfrb-CreERT2-GFP* or from *Pdgfrb-CreERT2-MHCII* were collected in ice-cold DMEM medium (Gibco by life technologies corporation by Thermo Fisher Scientific, Waltham, MA, USA; 31944021) supplemented with 1% penicillin–streptomycin (Sigma-Aldrich, St. Louis, MO, USA; P4333). Then, samples were further processed using the adult brain dissociation kit from Miltenyi Biotec (Bergisch Gladbach, Germany; 130-107-677) according to the manufacturer’s protocol to perform tissue digestion, homogenization and removal of remaining debris and red blood cells. The resulting single-cell suspension was cultured on collagen I-coated culture plates (BD Biosciences, San Diego, CA, USA; 354249) in endothelial cell medium as used before [39] for the first two passages. The medium was changed every two days. In passage three, medium was changed to pericyte medium (ScienCell Research Laboratories, Carlsbad, CA, USA; 1201). For passaging, cells were trypsinized using Trypsin–EDTA (Sigma-Aldrich, St. Louis, MO, USA; T3924) when at least 90% confluency was reached, and cells were split 1/4 in new coated wells. For in vitro recombination cells were treated with 300 Units TAT-CRE recombinase (Sigma-Aldrich, St. Louis, MO, USA; SCR508) for 6 h in pericyte medium. Experiments using pericytes in vitro were performed with cells from passage 5 to 7. 

### 4.7. Immunofluorescence Analysis of Pericytes

2 × 10^4^ pericytes were seeded on collagen I-coated cover slips and grown until they reached confluency. Subsequently, cells were fixed with 4% paraformaldehyde (PFA) for 20 min at room temperature (RT). After washing three times with phosphate-buffered saline (PBS), cells were permeabilized with 0.1% TritonX-100 in PBS for 10 min at RT and blocked with 5% bovine serum albumin in PBS for 60 min at RT. Thereafter, primary antibody incubation was performed for 60 min at RT in blocking solution, followed by three times washing with PBS and secondary antibody incubation for 60 min at RT. Last, coverslips were washed three times with PBS before mounting with Fluoromount-G™ mounting medium, with DAPI (Invitrogen by Thermo Fisher Scientific, Carlsbad, CA, USA; 00-4959-52). Immunofluorescence images were acquired using the BioRevo BZ-9000 (Keyence, Itasca, IL, USA), followed by image processing (brightness and contrast correction, background subtraction) with ImageJ 1.53f51 [40]. The following antibodies were used as primary antibodies: anti-α-SMA (abcam, Cambridge, UK; 197240); anti-desmin (abcam, Cambridge, UK; ab195177); anti-PDGFRβ (abcam, Cambridge, UK; 69506) and anti-CD31 (R&D systems, Abingdon, UK, AF3628). Secondary Cy-2 conjugated antibodies from dianova (Hamburg, Germany) were used for counterstaining.

### 4.8. Analysis of Pericyte Protein Expression In Vitro

To assess activation of pericytes in vitro we used T cell supernatant (TCS) of stimulated CD4^+^ T cells. For this purpose, CD4^+^ T cells were isolated from spleen and lymph nodes from 10–12-week-old C57BL/6 mice using immunomagnetic separation with CD4^+^-labelled MACS microbeads (Miltenyi Biotec, Bergisch Gladbach, Germany; 130-091-041) according to the manufacturer’s protocol. The isolated cells were cultured in T cell medium (IMDM medium (Gibco by life technologies corporation by Thermo Fisher Scientific, Waltham, MA, USA; 21980032) containing 10% FCS (Sigma-Aldrich, St. Louis, MO, USA; F7524), 50 µM 2-mercaptoethanol (Gibco by life technologies corporation by Thermo Fisher Scientific, Waltham, MA, USA; 31350-010) and 1% penicillin–streptomycin) in 96-well plates. T cells were either left unstimulated (control) or were stimulated with α-mouse CD3 monoclonal antibody (mAb) (4 µg/mL; clone 145-2C11, Biolegend, San Diego, CA, USA; 100302) and α-mouse CD28 mAb (4 µg/mL; clone 37.51, Biolegend, San Diego, CA, USA; 102116) for 72 h. Afterwards, TCS was collected and stored at −20 °C until use. To analyze the expression of different proteins on pericytes of the cerebral cortex, 2 × 10^4^ pericytes were seeded in 48-well plates coated with collagen I and then treated with either stimulated TCS or unstimulated TCS for 72 h. Thereafter, cells were detached using Accutase (Corning, NY, USA; 25-058-CI) and further processed for flow cytometric analysis. 

### 4.9. Analysis of Pericyte Protein Expression Ex Vivo

For analyzing protein expression of brain pericytes ex vivo under inflammatory conditions, brains of *Pdgfrb-CreERT2-GFP* EAE mice or non-immunized control mice were dissected at the day of disease maximum (day 15). For tissue digestion and removal of remaining debris and red blood cells, mouse cortices were further processed using the adult brain dissociation kit from Miltenyi according to the manufacturer’s protocol. The resulting single-cell suspension was collected in MACS buffer (PBS (Sigma-Aldrich, St. Louis, MO, USA; D8537), containing 1% FCS and 2 mM EDTA (Sigma-Aldrich, St. Louis, MO, USA; F5134)) and further processed for flow cytometric analysis.

### 4.10. Adhesion Assay

The static adhesion assay was performed as described before by Lowe & Raj 2015 [41]. For this, 4 × 10^4^ pericytes were seeded on collagen I-coated coverslips in a 24-well plate. Isolated CD4^+^ T cells from spleen and lymph nodes of 10–12-week-old C57BL/6 mice were stimulated with α-mouse CD3 mAb (4 µg/mL; clone 145-2C11, Biolegend, San Diego, CA, USA;100302) and α-mouse CD28 mAb (4 µg/mL; clone 37.51, Biolegend, San Diego, CA, USA); 102116) for 72 h or left unstimulated. Afterwards, T cells were labelled with the green fluorescent dye carboxyfluorescein succinimidyl ester (CFSE) (Thermo Fisher Scientific, Waltham, MA, USA; V12883). For the adhesion assay, 1 × 10^6^ T cells were added per well with pericytes and were allowed to attach for 60 min. Afterwards, coverslips were immersed in Hank’s balanced salt solution for five times. This step was repeated three times and then the coverslip was incubated in 4% PFA for 15 min at RT. Thereafter, the coverslips were mounted on microscope slides with DAPI counterstain and images were acquired using a Zeiss AxioObserver microscope (Jena, Germany). For each coverslip at least five images were taken and attached T cells were counted per image using ImageJ [40]. To normalize the results, the number of CD4^+^ T cells attached to a coated coverslip without pericytes was used as a negative control.

### 4.11. Analysis of CD4^+^ T Cell Responses In Vitro

CD4^+^ T cells were isolated from MOG_35–55_-transgenic (2D2) [42] or OVA_323–339_-transgenic (OT-II) [43] animals by immunomagnetic separation using CD4^+^-labelled MACS microbeads (Miltenyi Biotec, Bergisch Gladbach, Germany; 130-091-041) according to the manufacturer’s protocol and cultured in T cell medium. For analysis of T cell proliferation, T cells were labeled with the cell proliferation dye eFluor™ 670 (eBioscience by Thermo Fisher Scientific, Waltham, MA, USA; 65-0840-85) according to manufacturer’s instructions. Dendritic cells (DCs) were obtained from the spleens of 10–12-week-old C57BL/6 mice using CD11c^+^-labelled MACS microbeads (Miltenyi Biotec, Bergisch Gladbach, Germany; 130-125-835) according to the manufacturer’s protocol. Afterwards, 1 × 10^5^ T cells were co-cultivated with either 2 × 10^4^ TCS stimulated pericytes or 1 × 10^5^ DCs (used as positive control) loaded with distinct peptides for 72 h. For stimulation of OT-II T cells, cells were loaded with the OVA_323–339_ peptide (5 µg/mL). For stimulation of 2D2 T cells, cells were loaded with the NFM_15–35_ peptide (10 µM). After incubation, cells were collected using detachment via Accutase and processed for further analysis of T cell proliferation via flow cytometry.

For T cell activation marker analysis, 2 × 10^4^ pericytes were loaded with NFM_15–35_ peptide (10 µM) and co-cultured with 1 × 10^5^ 2D2 CD4^+^ T cells for 48 h. Then, cells were detached with Accutase and stained for flow cytometric assessment.

### 4.12. Isolation of Mononuclear Cells from the CNS

Mice were sacrificed under deep anesthesia by intra-cardiac perfusion with PBS. Brains and spinal cords were removed, and mononuclear cells were isolated as previously described [31]. For flow cytometric measurement of intracellular cytokines, isolated immune cells were restimulated with leukocyte activation cocktail (BD, Sparks, MD, USA; 550583) according to the manufacturer’s protocol for 4 h at 37 °C.

### 4.13. Flow Cytometry of Murine Immune Cells and Pericytes

All antibodies, if not stated otherwise, were obtained from Biolegend (San Diego, CA, USA). Surface marker staining was performed as previously described [44] and the following antibodies were used: α-mouse CD4 (clone: GK1.5), α-mouse CD8a (clone: 53-6.7), α-mouse CD11b (clone: M1/70), α-mouse CD45 (clone: 30-F11), α-mouse CD25 (clone: PC61), α-mouse CD69 (clone: H1.2F3), α-mouse PDGFRβ (clone: APB5), α-mouse MHC II (clone: M5/114.15.2), α-mouse ICAM-1/CD54 (clone: 3E2), α-mouse VCAM-1/CD106 (clone 429, eBioscience by Thermo Fisher Scientific, Waltham, MA, USA), α-mouse CD40 (clone: 3/23), α-mouse CD80 (clone: 16-10A1), and α-mouse CD86 (clone: GL-1). Intracellular staining of cytokines α-mouse IL-17A (clone: TC11-18H10.1) and α-mouse IFN-γ (clone: XMG1.2) was performed with BD Cytofix/Cytoperm™ Fixation/Permeabilization Solution Kit (BD Biosciences, San Diego, USA; 554714) according to the manufacturer’s protocol. For determination of the absolute cell counts, CountBright™ Absolute Counting Beads (Invitrogen by Thermo Fisher Scientific, Carlsbad, CA, USA; C36950) were used as recommended. Flow cytometric analysis was performed using a Gallios Flow Cytometer (Beckman Coulter, Krefeld, Germany) and a Cytoflex Flow Cytometer (Beckman Coulter, Krefeld, Germany). Results were analyzed with Kaluza 2.1 (Beckman Coulter, Krefeld, Germany) software.

### 4.14. Immunohistochemistry of EAE Tissue

Immunohistochemistry was performed as described before [44,45]. Mice were sacrificed under deep anesthesia by intra-cardiac perfusion with PBS followed by perfusion with 4% (*w*/*v*) PFA dissolved in PBS. Spinal cords and brains were removed and fixed in 4% PFA overnight. Before embedding in paraffin and sectioning in 3 µm slices, the spinal cord was cut into 7–10 transverse segments (3 mm thick) and coronal brain cuts were made. The sections were pretreated with citrate buffer (pH 6 or 9) in a steamer. Immunohistochemistry was then performed using the biotin–streptavidin peroxidase technique (Dako by Agilent Technologies, Santa Clara, CA, USA) with an automated immunostainer (AutostainerLink 48, Dako by Agilent Technologies, Santa Clara, CA, USA). The primary antibodies used were specific to Mac3 (clone M3/84; BD Pharmingen, San Jose, CA, USA) or CD3 (MCA, Serotec, Oxford, UK). 3,3′-diaminobenzidine (Dako by Agilent Technologies, Santa Clara, CA, USA) was used as color substrate and sections were mounted with Eukitt^®^ mounting medium (O. Kindler GmbH, Bobingen, Germany) after dehydration. Images were acquired with the microscope AxioObserver (Carl Zeiss, Jena, Germany) or BioRevo BZ-9000 (Keyence, Itasca, IL, USA) using the software AxioVision or BZ-II Analyzer (Keyence, Itasca, IL, USA).

### 4.15. Statistical Analysis

All results are shown as the mean ± SD unless stated otherwise. GraphPad Prism software (LA Jolla, CA, USA) was used to perform statistical analysis and visualize the results. Unpaired, two-sided Mann–Whitney test was used as a non-parametric test for two groups. Kruskal–Wallis Test was performed as a non-parametric test for three groups (one-way ANOVA). AT-EAE (Figure 3a) experiments were statistically analyzed using two-way ANOVA with Bonferroni’s post-test. *p* values < 0.05 were considered as statistically significant. *, *p* ≤ 0.05; **, *p* ≤ 0.01; ***, *p* ≤ 0.001; ****, *p* ≤ 0.0001.

## Data Availability

The datasets used/or analyzed during the current study are available from the corresponding author on reasonable request.

## Supplementary Materials

The following supporting information can be downloaded at: https://www.mdpi.com/article/10.3390/ijms232113081/s1.

## Abbreviations

α-SMA	alpha-smooth muscle actin
AD	Alzheimer´s disease
APC	antigen presenting cell
AT-EAE	adoptive transfer EAE
BBB	blood–brain barrier
CADASIL	cerebral autosomal dominant arteriopathy with subcortical infarcts
CNS	central nervous system
ctrl	control
DC	dendritic cell
EAE	experimental autoimmune encephalomyelitis
ICAM	intracellular adhesion molecule
mAb	anti-mouse antibody
MFI	mean fluorescence intensity
MHC	major histocompatibility complex
MOG	myelin oligodendrocyte glycoprotein
MS	Multiple Sclerosis
OVA	ovalbumin
PBS	phosphate-buffered saline
PDGFR	platelet-derived growth factor receptor
PFA	paraformaldehyde
RT	room temperature
TCS	T cell supernatant
VCAM	vascular cell adhesion molecule
