# Supplementary material for: CNS Pericytes Modulate Local T Cell Infiltration in EAE
Source: Int J Mol Sci. 2022 Oct 28;23(21):13081. doi: 10.3390/ijms232113081 (PMC9658756; doi:10.3390/ijms232113081)
Supplement: Supplementary file 1 [file ijms-23-13081-s001.zip › ijms-1914247-supplementary.pdf]

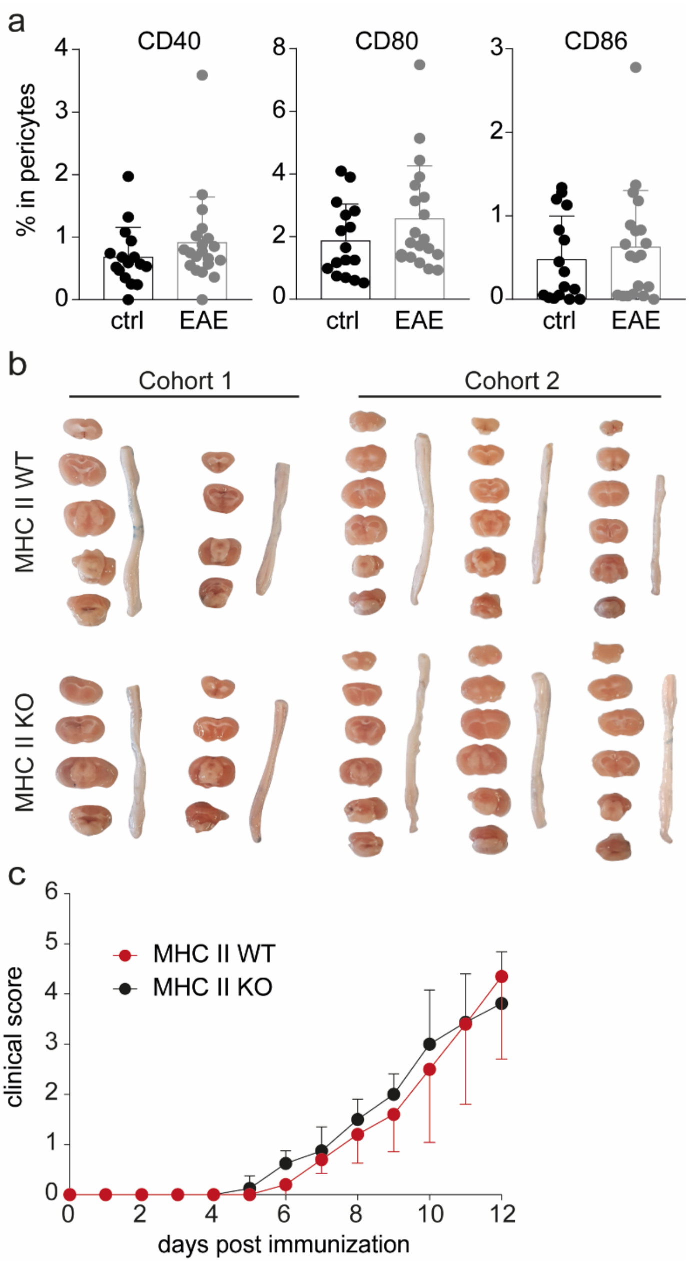

Supplementary Figure S1. Expression of costimulatory molecules on CNS pericytes from EAE animals was not altered and MHC II KO did not influence BBB integrity. **(a)** Bar graphs depict frequencies of pericytes expressing the costimulatory molecules CD40, CD80, and CD86. At disease maximum, pericytes were isolated from the CNS of *Pdgfrb-CreERT2-GFP* animals immunized with $MOG_{35\text{-}55}$ (EAE; $n = 20$) in comparison to non-immunized mice (ctrl; $n = 16$). Data are shown as mean ± SD. Single dots represent individual values of single mice. **(b)** Analysis of coronal brain slices and spinal cords of MHC II WT (upper) and MHC II KO (lower) animals from the *Pdgfrb-CreERT2-MHCII* mouse line after i.v. injection of Evan's blue dye of two independent cohorts are depicted. **(c)** MCH II KO did not affect disease outcome of active EAE. EAE disease course of MHC II WT and MHC II KO animals is shown. Disease courses MHC II WT mice ($n = 5$) compared to MHC II KO mice ($n = 4$) are shown as mean group scores ± SD over time. Data shown are representative data from one experiment out of three independent experiments.

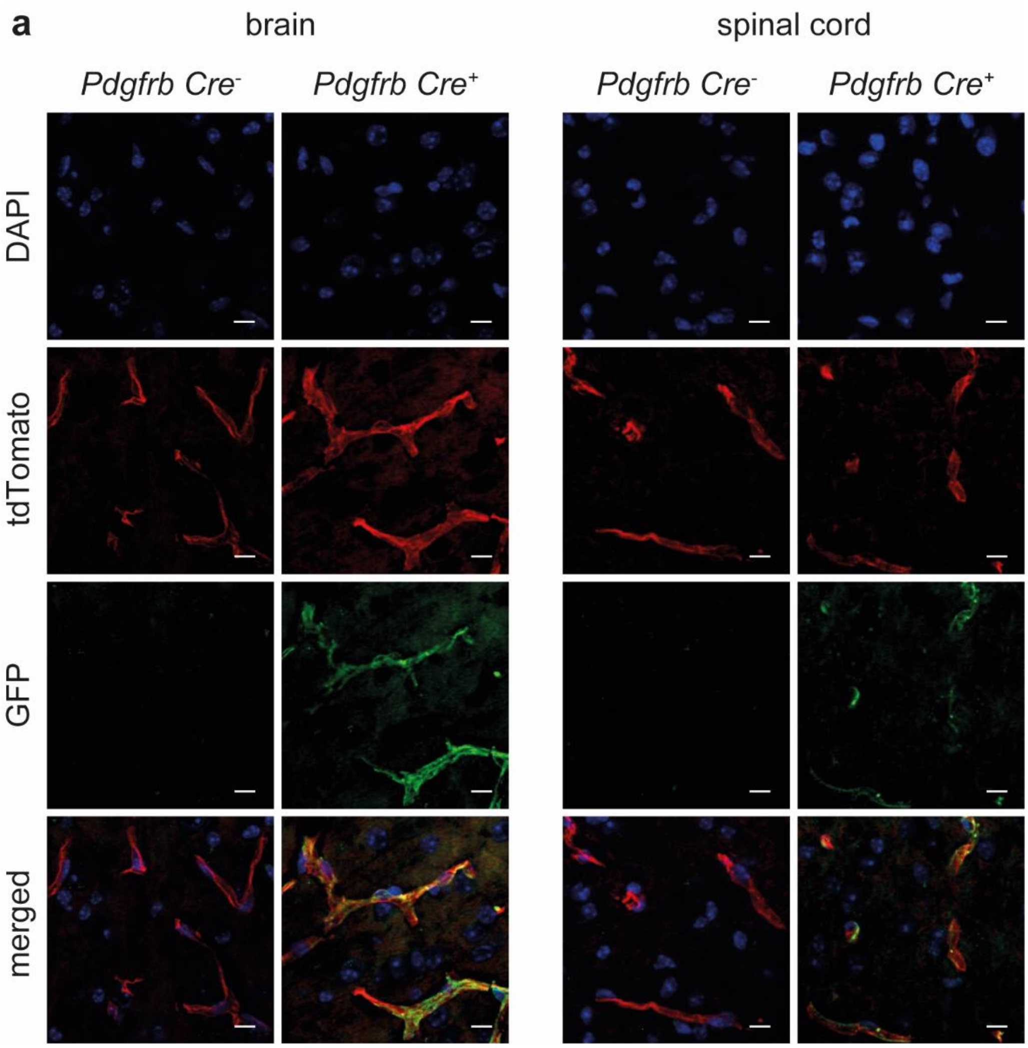

Supplementary Figure S2. Only Cre+ littermates of *Pdgfrb-Cre-GFP* mice display GFP expression. **(a)** Immunohistochemistry analysis was performed with of 10 µm-thick cryosections of brains and spinal cords from Cre+ and Cre- *Pdgfrb-Cre-GFP* animals. Only representative examples of the nuclei staining (DAPI), tdTomato signals (related to *Pdgfrb* promotor), and GFP signals (related to Cre recombination under the *Pdgfrb* promotor) are shown (scale bar = 10 µm).
